# Combined Use of a Ramped Position Pillow and Bean Bag in a Morbidly Obese Patient Undergoing Robotic Pelvic Surgery: A Case Report

**DOI:** 10.1002/ccr3.72281

**Published:** 2026-03-17

**Authors:** Evangelia Sgouta, Fotios Vlahos, Iosifina Giannakikou

**Affiliations:** ^1^ National Kapodistrian University of Athens Medical School 3rd Year Medical Student Athens Greece; ^2^ University of Milano‐Bicocca Milano‐Bicocca Medical School 4th Year Medical Student Milan Italy; ^3^ Hygeia Hospital, Department of Anesthesia and Critical Care Medicine Consultant Anesthesiologist and Critical Care Physician Athens Greece

**Keywords:** anesthesia, ergonomics, morbid, obesity, patient safety, robotic surgery procedures

## Abstract

Vacuum bean bags improve patient stability and reduce pressure‐ and nerve‐related injuries during pelvic robotic surgery. In morbidly obese patients, the ramped position enhances airway management and ventilation, but maintaining it throughout surgery can be challenging. We report a case in which a ramped pillow was kept atop a vacuum bean bag for the entire procedure, eliminating the need for repositioning after intubation and before extubation. This approach minimized airway and line displacement, reduced staff strain, and preserved optimal oxygenation and ventilation during emergence and post‐extubation. The case highlighted the importance of individualized, tailored positioning strategies for morbidly obese patients undergoing prolonged robotic procedures.

## Key Clinical Message

Maintaining a ramped pillow atop a vacuum bean bag during robotic pelvic surgery in morbidly obese patients enhances airway management, ventilation, and stability, while preventing loss of optimal positioning and reducing staff strain. This novel, low‐cost strategy emphasizes multidisciplinary planning and tailored positioning to improve safety, efficiency, and ergonomics.

## Graphical abstract

30° ramped sniffing position using a ramped pillow on a bean bag facilitating airway axis alignment in a morbidly obese patient.

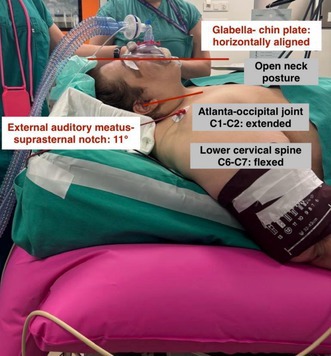

**Abbreviations**
BMI: body mass index
C: cervical
CPAP: continuous positive airway pressure
ETCO₂: end tidal carbon dioxide
FiO₂: fraction of inspired oxygen
FRC: functional residual capacity
HELP: head‐elevated laryngoscopy position
PIP: peak inspiratory pressure
O₂: oxygen
PaCO₂: arterial partial pressure of carbon dioxide
PaO₂: arterial partial pressure of oxygen
PEEP: positive end‐expiratory pressure
Pplat: plateau pressure
RR: respiratory rate
SaO_2_
: arterial oxygen saturation
SpO₂: peripheral oxygen saturation
TV: tidal volume
V/Q: ventilation‐perfusion

## 1 Introduction

Morbid obesity increases the risk of difficult airway management, aspiration, and postoperative respiratory compromise, risks that can be mitigated by the head‐elevated laryngoscopy position (HELP) or ramped position, which improve airway alignment and ventilation. Pelvic robotic surgery in steep Trendelenburg further exacerbates respiratory compromise and introduces risks of patient slippage, pressure injuries, and nerve damage, often necessitating vacuum bean bags to enhance stability and reduce pressure‐related complications. Although the ramp is commonly removed after intubation, doing so forfeits its benefits during emergence and extubation, and repositioning it atop the vacuum bean bag at the end of surgery is technically challenging due to patient weight and friction. This case demonstrates a practical method of maintaining the HELP pillow on a vacuum bean bag throughout the procedure, optimizing airway management, ventilation, patient stability, and staff ergonomics in a morbidly obese patient. To our knowledge, this combined approach in robotic pelvic surgery is unique, as it has not been previously reported, particularly in a patient with a body mass index (BMI) of 55 kg/m^2^. Informed written consent was obtained from the patient for publication, and this report complies with the 2013 CARE guidelines.

## 2 Case History/Examination

A 56‐year‐old woman with class III morbid obesity (180 kg, 180 cm; BMI 55 kg/m^2^ after preoperative weight loss) underwent robotic hysterectomy with bilateral salpingo‐oophorectomy. Her comorbidities included hypertension (olmesartan, amlodipine, bisoprolol), hyperlipidemia (rosuvastatin), type 2 diabetes mellitus (metformin, semaglutide), grade 3 fatty liver disease, gastroesophageal reflux, diaphragmatic hernia, and obstructive sleep apnea (minimum nocturnal peripheral oxygen saturation [SpO_2_] 79%) managed with automated continuous positive airway pressure (CPAP).

On examination, she had a Mallampati class I airway, neck circumference of 48 cm, large breasts, and central adiposity. Laboratory tests and pulmonary function were normal, with no evidence of restrictive disease, and room‐air SpO_2_ was 96%. Preoperative weight reduction and respiratory rehabilitation were recommended.

## 3 Differential Diagnosis, Investigations and Treatment

The patient was positioned on a 30° HELP pillow atop a 90 cm‐wide deflated vacuum bean bag on a bariatric table. Prior to induction, the breasts and panniculus were taped caudally and laterally to reduce thoracic compression. Pre‐induction SpO_2_ was 94%, improving to 100% after preoxygenation with 5 cm H_2_O CPAP. General anesthesia was induced with propofol, lidocaine, fentanyl, and rocuronium, and the trachea was intubated uneventfully using video laryngoscopy with a 7.5‐mm endotracheal tube. Oxygen saturation remained stable throughout apnea, preventing measurement of safe apnea time. Invasive arterial blood pressure monitoring was established after intubation.

In the ramped position, airway pressures were peak inspiratory pressure (PIP) 34 cm H_2_O, plateau pressure (Pplat) 24 cm H_2_O, and positive end‐expiratory pressure (PEEP) 10 cm H_2_O. Arterial blood gas analysis showed a partial pressure of oxygen (PaO_2_) of 266 mmHg and an arterial oxygen saturation (SaO_2_) of 99.9% on a fraction of inspired oxygen (FiO_2_) of 0.5. The partial pressure of carbon dioxide (PaCO_2_) was 46 mmHg, with an end‐tidal carbon dioxide (ETCO_2_) of 42 mmHg while the patient was ventilated in pressure‐controlled volume‐guaranteed mode with a tidal volume (TV) of 400 mL and a respiratory rate (RR) of 10 breaths/min (Table 1, column A).

**TABLE 1:** Airway pressures, arterial blood gas values, ventilation settings, and vital signs at key perioperative time points.

	Column A	Column B	Column C
	Post‐induction, ramped 30°, supine	Pneumoperitoneum 11 mmHg, ramped 30°, trendelenburg 27°	Post‐extubation, ramped 30°, supine
*Airway pressures*			
PIP (cmH_2_O)	34	34	
Pplat (cmH_2_O)	24	25	
PEEP (cmH_2_O)	10	10	
CPAP (cmH_2_O)			Automated
*Arterial blood gases*			
PaO_2_ (mmHg)	266	82.8	183
FiO_2_ (%)	50	60	
SaO_2_ (%)	99.9	93.7	99.5
O_2_ (L/min)			6
PaCO_2_ (mmHg)	46	59.2	45.7
*Ventilator settings*			
TV mL	400	400	Spontaneous
RR breaths/min	10	14	Spontaneous
*Vital signs*			
SpO_2_ (%)	100	93	99
ETCO_2_ (mmHg)	42	45	Present

*Note:* Column A: post‐induction, ramped 30°, supine; Column B: ramped 30°, Trendelenburg 27°, pneumoperitoneum 11 mmHg; Column C: post‐extubation, ramped 30°, supine. Abbreviations: CPAP, continuous positive airway pressure; ETCO_2_, end tidal carbon dioxide; FiO_2_, fraction of inspired oxygen; O_2_, oxygen; PaCO_2_, arterial partial pressure of carbon dioxide; PaO_2_, arterial partial pressure of oxygen; PEEP, positive end‐expiratory pressure; PIP, peak inspiratory pressure; Pplat, plateau pressure; RR, respiratory rate; SaO_2_, arterial oxygen saturation; SpO_2_, peripheral oxygen saturation; TV, tidal volume.

The patient was then placed in steep Trendelenburg (27°) and lithotomy using the vacuum bean bag, with arms tucked. Positioning was technically challenging, and the surgical team ultimately removed the panniculus tape prior to draping to optimize access and maintain sterility. Unlike standard practice, where the HELP pillow is removed after intubation, it was retained throughout surgery to preserve airway alignment and facilitate postoperative ventilation.

Following Trendelenburg positioning and pneumoperitoneum at 11 mmHg, airway pressures remained essentially unchanged, with a PIP of 34 cm H_2_O and a Pplat of 25 cm H_2_O. Ventilation was maintained with TV 400 mL, RR 14/min, and PEEP 10 cm H_2_O, with intermittent automated recruitment maneuvers performed hourly. Oxygenation remained adequate, with mean PaO_2_ 82.8 mmHg and SaO_2_ 93.7% on FiO_2_ 0.6, and permissive hypercapnia (mean PaCO_2_ 59.2 mmHg, ETCO_2_ 45 mmHg) (Table 1, column B). Total operative time was 213 min.

At the conclusion of surgery, the patient was returned to a supine ramped position and extubated without complications. Immediately post‐extubation, SpO_2_ was 99% on 10 L/min oxygen (O_2_) via an anesthesia face mask with 5 cm H_2_O CPAP.

## 4 Outcome and Follow up

She was transferred to the post‐anesthesia care unit in a semi‐recumbent position on automated CPAP with 6 L/min supplemental O_2_, maintaining SpO_2_ 99%. Arterial blood gas analysis showed PaO_2_ 183 mmHg, PaCO_2_ 45.7 mmHg, and SaO_2_ 99.5% (Table 1, column C).

The patient was subsequently transferred to the surgical ward with instructions to keep the head of the bed elevated at least 30°, wean supplemental O_2_ to maintain SpO_2_ above 94%, and continue CPAP during sleep. Supplemental O_2_ was discontinued 6 h postoperatively, and she ambulated the same day. Postoperative pain was controlled with paracetamol and non‐steroidal anti‐inflammatory drugs; no rescue opioids were required. No episodes of desaturation or respiratory complications occurred, and no postoperative arterial blood gas analysis or chest radiography was performed. The patient reported improved breathing, no positional neck or back discomfort, and was discharged on postoperative day two.

## 5 Discussion

Patients with class III obesity present significant airway and respiratory challenges, including difficult airway management and impaired oxygenation and ventilation, particularly during induction and post‐extubation [1, 2, 3]. Excess adipose tissue around the neck and face increases the risk of difficult mask ventilation and intubation, with neck circumference and Mallampati score serving as key predictors [1, 2]. Obesity also increases O_2_ consumption due to higher metabolic demands and elevates work of breathing [2]. Reduced chest wall compliance and lung volumes lower functional residual capacity (FRC) below closing capacity, leading to ventilation–perfusion (V/Q) mismatch, diminished O_2_ reserves, and rapid desaturation [2, 4]. These pathophysiologic effects are further amplified during robotic pelvic surgery, as pneumoperitoneum and steep Trendelenburg exacerbate reductions in lung compliance, increase airway resistance, and worsen V/Q mismatch, thereby increasing the risk of hypoxemia and hypercarbia. In addition, steep Trendelenburg can promote airway edema and make extubation more challenging [2, 3, 4, 5]. Elevated intra‐abdominal pressure, higher prevalence of hiatal hernia, and increased residual gastric volume further increase aspiration risk [2]. Collectively, morbid obesity, pneumoperitoneum, and steep Trendelenburg substantially increase perioperative airway and respiratory challenges [2, 4].

**FIGURE 1:**
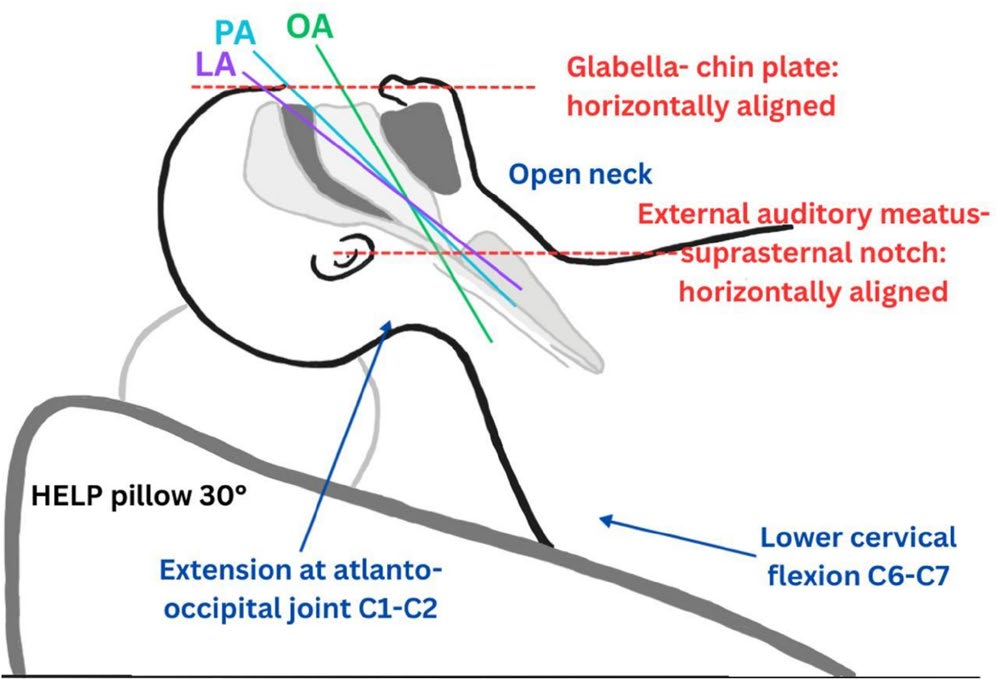
Schematic of the ideal sniffing position in a morbidly obese patient on a 30° ramp with a HELP pillow. The oral, pharyngeal, and laryngeal axes are aligned, with the glabella–chin and external auditory meatus–suprasternal notch planes horizontal, and atlanto‐occipital (C1–C2) extension plus lower cervical (C6–C7) flexion. HELP, head‐elevated laryngoscopy position; C, cervical; OA, oral axis; PA, pharyngeal axis; LA, laryngeal axis.

Optimal intubation is classically achieved in the sniffing position, aligning the oral, pharyngeal, and laryngeal axes via atlanto‐occipital (C1–C2) extension and lower cervical (C6–C7) flexion, creating an open‐neck configuration with the face parallel to the ceiling (Figure 1) [1]. This alignment establishes a linear path from the mouth to the glottis, improving laryngoscopic visualization, increasing intubation success, and reducing intubation time [1, 4]. In morbidly obese patients, the ramped position achieves similar alignment by horizontally orienting the glabella–chin and external auditory meatus–suprasternal notch planes, enhancing preoxygenation, safe apnea time, laryngoscopic view, FRC, and ventilation while reducing aspiration risk [1, 2, 4, 6]. Thoracic and diaphragmatic pressure can be further relieved by taping the breasts and panniculus downward and laterally toward the lateral thorax and thighs, respectively [7]. Positional stability in steep Trendelenburg is supported by a vacuum bean bag, which evenly distributes weight, prevents displacement, and reduces pressure injuries and peripheral nerve damage.

Removing the HELP pillow after intubation, as is standard practice to improve surgical access, increases the risk of airway or line displacement and complicates repositioning at the conclusion of surgery due to patient weight and the anti‐sliding properties of the vacuum bean bag [1, 4]. Maintaining the HELP pillow throughout surgery minimizes these complications, reduces staff strain by eliminating the physical effort of lifting the head and trunk, preserves optimal postoperative respiratory function, and streamlines transitions from induction to emergence, thereby shortening anesthesia management time [1].

This case illustrates a simple, effective, and reproducible strategy for a clinically relevant problem: intentionally maintaining a ramped pillow atop a vacuum bean bag throughout surgery to optimize airway alignment and respiratory mechanics while allowing secure positioning in steep Trendelenburg. The approach is practical, easily implemented, and requires no specialized or expensive equipment.

Limitations include generalizability, as this report describes a single case. Increased bed height—up to 40 cm depending on the combined thickness and compressibility of the pillow and bean bag—may interfere with robotic arm docking or staff access [1]. These challenges can be addressed by offsetting the operating table to prevent interference between robotic arms and surgical lights and by using step platforms to facilitate staff access. Reduced contact with the bean bag may increase the risk of patient slippage, which can be minimized by custom‐molding the bag around both patient and pillow and using shoulder braces for additional stabilization [4]. To cover both the patient and HELP pillow, the uncompressed vacuum bean bag must be suspended from the head of the table so it supports both structures when suctioned into position; this is feasible in the lithotomy position when the bag extends only to the sacrum. Alternatively, the pillow could be placed and secured beneath the bean bag; however, this approach was not trialed by the team. Although soft, compressible materials are commonly used for ramping, reduced surface contact with the bean bag can increase the risk of pressure injuries and should be mitigated with additional padding. Maintaining the ramped position may not always be feasible, as the associated mild lumbar flexion can limit surgical exposure and compromise operative conditions. Lumbar flexion may also increase sacral pressure, potentially contributing to postoperative back pain or pressure‐related injury. Feasibility should therefore be assessed on a case‐by‐case basis, considering patient habitus, surgical requirements, and the specific robotic platform. Maintaining the ramped position may be particularly advantageous in patients with severe obesity, obstructive sleep apnea, increased aspiration risk, or anticipated difficulty with repositioning after prolonged robotic procedures. Conversely, this approach may be inappropriate when ramping compromises surgical exposure, interferes with robotic docking, or creates unacceptable ergonomic challenges. Clear criteria are needed to define patient selection and procedural limitations.

**FIGURE 2:**
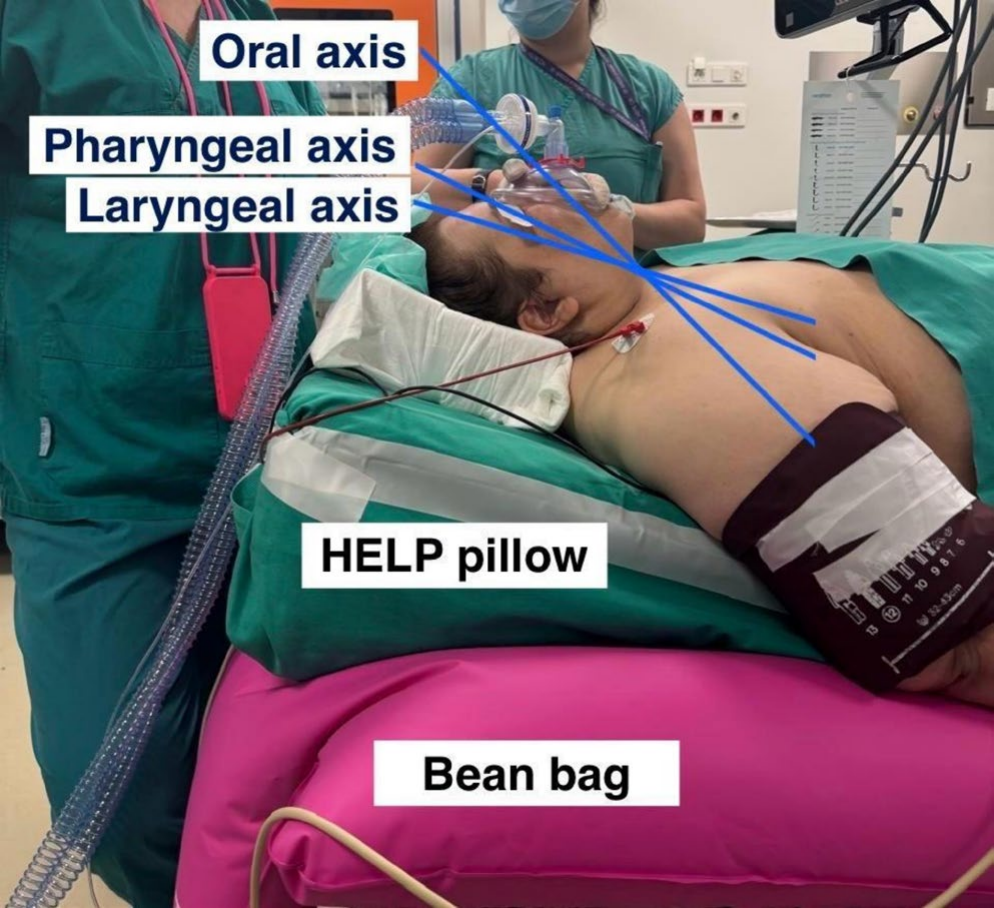
Clinical photograph demonstrating proper alignment of the oral, pharyngeal, and laryngeal axes. The patient's breasts were taped caudally and laterally to the thorax to reduce thoracic compression; this is not visible due to surgical drapes. The uncompressed vacuum bean bag and HELP pillow are visible. HELP, head‐elevated laryngoscopy position.

**FIGURE 3:**
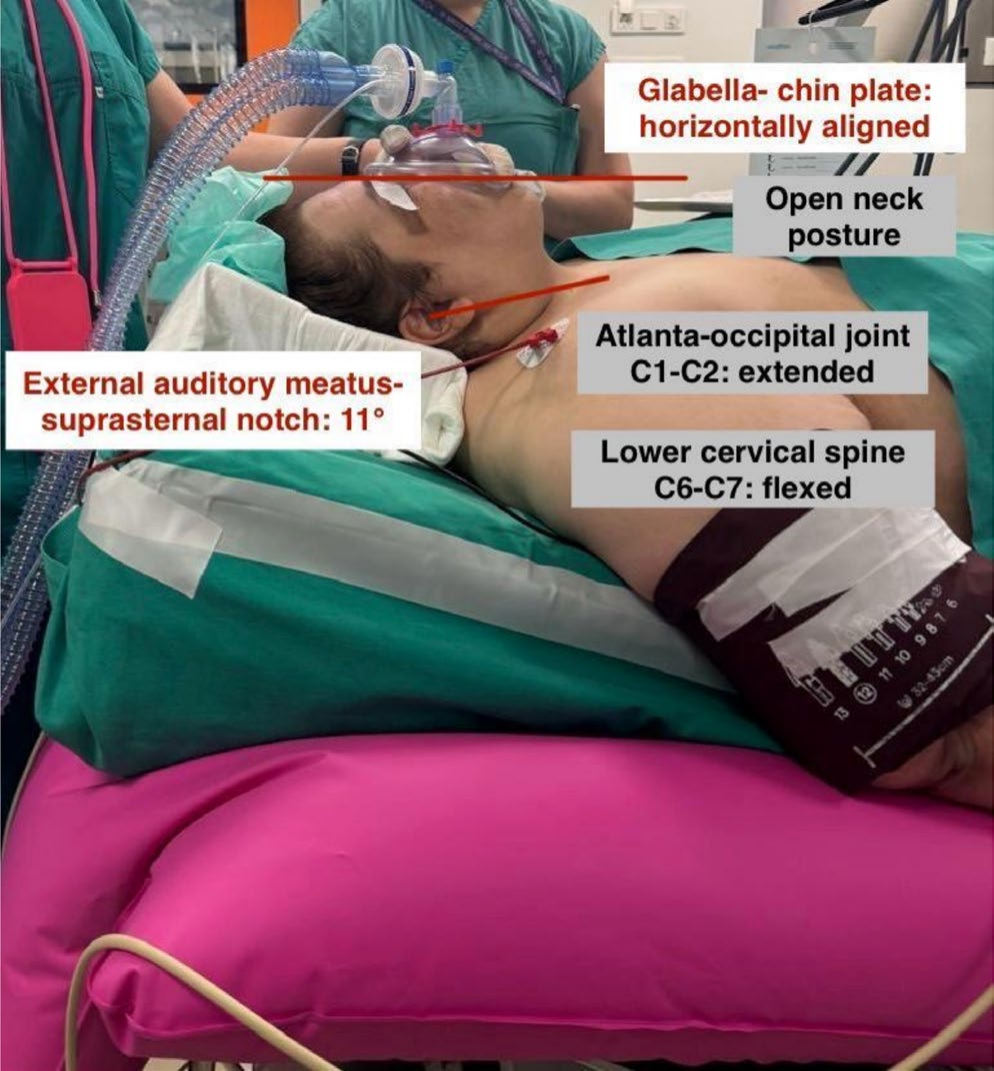
Clinical photograph demonstrating horizontal glabella–chin alignment with an 11° external auditory meatus–suprasternal notch angle. This configuration produces atlanto‐occipital (C1–C2) extension and lower cervical (C6–C7) flexion, resulting in an open‐neck position. C, cervical.

In our patient, the ramped position was maintained throughout surgery due to concerns regarding rapid desaturation after induction and extubation, as well as the anticipated difficulty of repositioning the pillow at the conclusion of surgery. During surgery, PaO_2_ and SaO_2_ fell despite increased FiO_2_, while PaCO_2_ rose with minimal ETCO_2_ change, reflecting V/Q mismatch, increased dead space, and impaired alveolar ventilation from Trendelenburg positioning, pneumoperitoneum, and obesity. Postoperatively, oxygenation and ventilation rapidly recovered on CPAP with supplemental oxygen, demonstrating the reversibility of these intraoperative impairments. This configuration also optimized upper airway alignment throughout the procedure (Figures 2, 3). The patient's history of gastroesophageal reflux, hiatal hernia, and recent glucagon‐like peptide‐1 receptor agonist use further increased aspiration risk, making this positioning particularly advantageous. Although taping the breasts and panniculus during preoxygenation improved respiratory mechanics, the panniculus tape was later removed by the surgical team to optimize surgical access, highlighting the importance of coordinated multidisciplinary planning. Because no preoperative simulation of the final operative configuration was performed, intraoperative adjustments to table position and overhead lighting were required to accommodate robotic arm docking. These observations underscore the importance of individualized preoperative simulation and multidisciplinary planning to ensure patient safety and optimal ergonomics [2, 3, 4, 5].

Morbid obesity significantly complicates airway management and perioperative respiratory physiology, particularly during robotic surgery in steep Trendelenburg. This report describes a morbidly obese patient undergoing prolonged robotic pelvic surgery in whom a ramped pillow was intentionally maintained atop a vacuum bean bag throughout the procedure. This configuration optimized airway management, ventilation, and patient positioning without compromising surgical exposure or safety. This approach is novel not for new technology, but for deliberately combining two established positioning aids to address a clinically relevant challenge, offering a low‐cost, potentially effective strategy. Although logistical challenges may arise, these can be mitigated through careful preoperative planning and appropriate equipment adjustments. Although a single case, it provides actionable insights beyond prior studies on ramped positioning or bean bags, highlighting thorough preoperative evaluation and individualized risk–benefit assessment.

## Author Contributions

**Evangelia Sgouta:** conceptualization, data curation, investigation, validation, visualization, writing – original draft, writing – review and editing. **Fotios Vlahos:** conceptualization, data curation, investigation, validation, visualization, writing – original draft, writing – review and editing. **Iosifina Giannakikou:** project administration, supervision, validation, writing – review and editing.

## Funding

The authors have nothing to report.

## Ethics Statement

The authors have nothing to report.

## Consent

Written informed consent was obtained from the patient for publication of this case report.

## Conflicts of Interest

The authors declare no conflicts of interest.

## Data Availability

Non‐applicable.
